# Musculoskeletal Pain Among Eye Care Professionals

**DOI:** 10.7759/cureus.39403

**Published:** 2023-05-23

**Authors:** Abdulaziz Al Taisan, Ali E Al Qurainees, Omar M AL Sowayigh, Munthir A Al Owayfir

**Affiliations:** 1 College of Medicine, King Faisal University, Al-Ahsa, SAU; 2 Ophthalmology, Armed Forces Hospital Southern Region, Khamis Mushait, SAU

**Keywords:** musculoskeletal pain, back, neck, ophthalmology, pain

## Abstract

Purpose

The purpose of this study is to investigate musculoskeletal pain among eye care professionals.

Methods

This cross-sectional study was conducted using an electronic detailed questionnaire through Google Forms. The structured questionnaire was distributed through various social media platforms targeting eye care professionals. The study included currently practicing ophthalmologists (Consultants, Specialists, Residents), optometrists, and orthoptists.

Results

A total of 514 eye care professionals participated in the study. The majority were younger than 30 years old (43.8%), with more than half being males (51.8%) and ophthalmologists (55.2%). The prevalence of eye care professionals who were suffering from musculoskeletal pain was 66.7%. The prevalence was significantly higher among females (76.2%) and those over 50 years old (71.4%). Sixty-eight point three percent (68.3%) of participants who don’t do running exercises and 92.2% of those with comorbidities suffer from pain. The prevalence of pain among eye care professionals who every week examine more than 150 patients is 72.4%, perform more than 20 surgeries is 85.7%, and conduct more than 20 laser treatment sessions is 100%.

Conclusion

Musculoskeletal pain is highly prevalent among eye care professionals. This is especially true among females and older adults (>50 years). Among different exercises, running is most protective against musculoskeletal pain. Comorbidities contribute significantly to developing pain.

## Introduction

Back and neck pains are common problems among the adult population [1,2]. This is also true among medical professionals [3-5], especially ophthalmologists and optometrists [6]. This is not strange considering eye care professionals adopt ergonomically awkward sitting positions along with repetitive tasks which strain their bodies [5,6]. The current prevalence of neck and upper back pain among eye care professionals in Saudi Arabia, as reported by a single-tertiary hospital study, is 70% [3].

Ophthalmologists have adopted different approaches to this problem, some have used analgesics [5] and others have used physical exercises as a method to prevent or reduce pain [3]. While it has been shown that physical exercises are beneficial for such pain [3], this has not been adequately investigated. So, we aim to investigate more types of musculoskeletal pain among a larger population of eye care professionals.

## Materials and methods

Study design and participants

This is a descriptive cross-sectional study that was conducted using an electronic detailed questionnaire through Google Forms. The structured questionnaire consisting of 36 questions was distributed through various social media platforms, including WhatsApp, Twitter, and Telegram targeting eye care professionals in Saudi Arabia. The study included practicing ophthalmologists (consultants, specialists, residents), optometrists, and orthoptists. All individuals who were not currently practicing or retired were excluded. The data variables that were collected were age, sex, occupation, BMI, city/region, exercise routine and type, working pattern, pain analysis, physical stress level, and pain treatment methods used.

The first section of the questionnaire included socio-demographic characteristics (age in years, sex, occupation, BMI), history of trauma, comorbidities, and surgeries related to neck, lower or upper back, wrist, and hand pain. Exercising regularly as yes or no and if yes, participants were asked about the type of exercise they perform. Respondents were asked about the number of patients seen per week, and ophthalmologists were asked about the number of surgeries performed per week and laser sessions per week. They were also asked about physical stress. The second section assesses the frequency and severity of the pain. Participants were asked about the presence of pain, the relation of this pain with work, and any treatment taken for pain.

The questionnaire was distributed to 10 faculty members of King Faisal University, College of Medicine to ensure the clarity and content of the questionnaire. The questionnaire was open for responses for five months from February to July 2021. After collecting the data through Google Forms, it was exported to Microsoft Excel (Microsoft Corporation, Redmond, WA) to process the information and encode open variables. Improvements were made regarding the logic of the answers. Some types of exercises were grouped as Other (such as volleyball, football, swimming, yoga, etc.). 

Statistical analysis

Categorical data were presented using numbers and percentages while continuous data were summarized using mean and standard deviation. The frequency of neck, lower or upper back, and wrist and hand pain were compared with different characteristics by using the chi-square test or independent sample t-test. Significant results generated between comparisons were then placed in a multivariate regression model to determine the independent predictors associated with neck, lower or upper back, and wrist and hand pain where the odds ratio as well as the 95% confidence interval were also being reported. The p-value of 0.05 was considered statistically significant. The data were analyzed using Statistical Packages for Social Sciences (SPSS) version 26 (Armonk, NY: IBM Corp.).

Ethical considerations

This study was conducted upon the approval of the research ethics committee of the College of Medicine, King Faisal University. All collected data are confidential and consent was obtained from all participants.

## Results

A total of 514 eye care professionals participated in the study. Table 1 demonstrates the socio-demographic characteristics of the participants. The most common age group was less than 30 years old (43.8%) with more than half being males (51.8%) while 45.3% had less than five years in practice. Furthermore, nearly 60% were living in the central region with 43.6% being optometrists and 40.9% being main surgeons. The proportion of participants who were having regular exercise was 38.3%. Fifty-two point one percent (52.1%) had normal BMI while 26.7% were overweight.

**Table 1:** Socio-demographic characteristics of eye care professionals (n=514)

Study variable	N (%)
Age group in years	
<30 years	225 (43.8%)
30 – 50 years	219 (42.6%)
>50 years	70 (13.6%)
Gender	
Male	266 (51.8%)
Female	248 (48.2%)
Years in practice	
<5 years	233 (45.3%)
5 – 10 years	112 (21.8%)
11 – 15 years	65 (12.6%)
16 – 20 years	33 (06.4%)
>20 years	71 (13.8%)
Residence region	
Central region	297 (57.8%)
Eastern region	118 (23.0%)
Western region	76 (14.8%)
Northern region	07 (01.4%)
Southern region	16 (03.1%)
Occupation	
Optometrist	224 (43.6%)
Main surgeon	210 (40.9%)
Assistant surgeon	74 (14.4%)
Orthoptist	06 (01.2%)
Regular exercise	
Yes	197 (38.3%)
No	317 (61.7%)
BMI level	
Underweight (<18.5 kg/m2)	47 (09.1%)
Normal (18.5 – 24.9 kg/m2)	268 (52.1%)
Overweight (25 – 29.9 kg/m2)	137 (26.7%)
Obese (≥30 kg/m2)	62 (12.1%)

**Figure 1:**
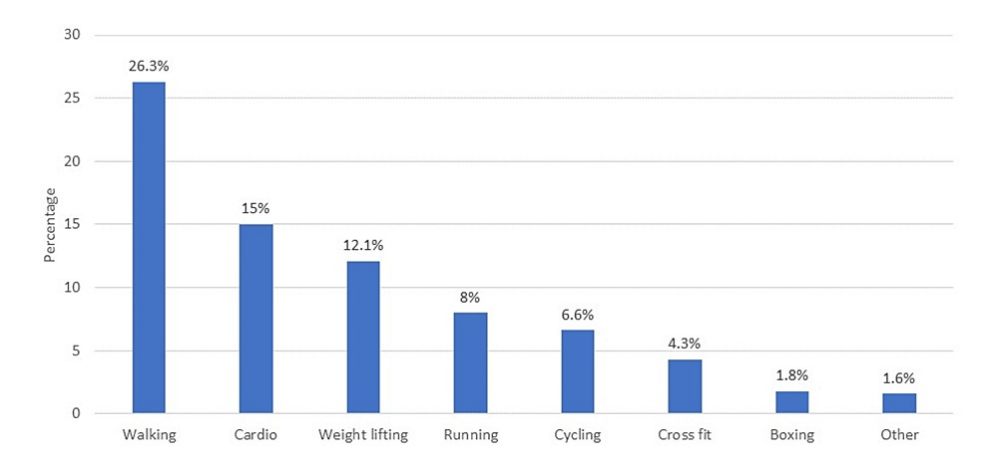
Type of regular exercise performed by eye care professionals

Figure 1 shows the type of regular exercise performed by eye care professionals, which demonstrates that the most common exercise performed by the respondents was walking.

**Figure 2:**
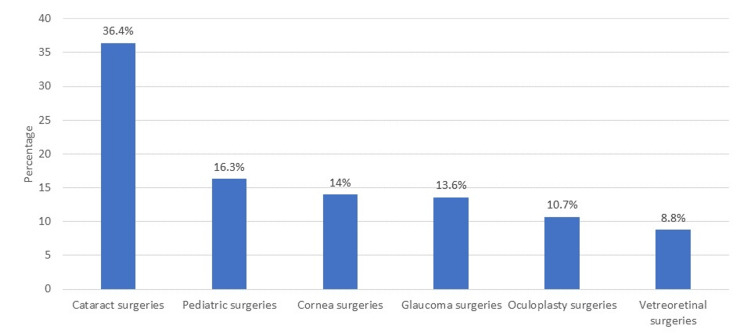
Types of surgeries performed by eye care professionals

In Figure 2, the most performed type of surgery was cataract surgery (36.4%), followed by pediatric surgeries (16.3%) and cornea surgeries (14%) while the least performed were vitreoretinal surgeries (8.8%).

Table 2 describes the previous history of neck, lower or upper back, wrist, and hand pain. We found that the prevalence of respondents with a previous history of trauma related to neck, lower or upper back, and wrist and hand pain was 17.9% while the prevalence of comorbidities related to pain was 20%. Furthermore, the proportion of respondents with a previous history of surgeries related to trauma was 3.3%. Nearly one-third (32.7%) of the eye care professionals examined 51-100 patients per week while 24.3% indicated that the average number of surgeries performed per week was less than five cases with a similar proportion (<5 cases) performed for laser treatment session per week (28.6%). Nearly half of the respondents (50.2%) reported work-related pain. The most used method for the treatment of pain was oral medicine (32.1%) followed by physiotherapy (21.8%). Additionally, the mean score of physical stress level was 5.74 (SD 2.11) out of 10 points.

**Table 2:** Previous history of neck, lower or upper back, and wrist and hand pain and its relation to practice (n=514)

Variables	N (%)
History of trauma related to the neck, lower or upper back, wrist, and hand pain?	
Yes	92 (17.9%)
No	422 (82.1%)
Comorbidities related to neck, upper or lower back pain, and wrist and hand pain	
Yes	103 (20.0%)
No	411 (80.0%)
Previous history of surgeries related to trauma	
Yes	17 (03.3%)
No	80 (15.6%)
Feel more pain when working as a	
Main surgeon	74 (14.4%)
Assistant surgeon	93 (18.1%)
Equal severity of pain in both	60 (11.7%)
No pain experienced	80 (15.6%)
I don't work as a main surgeon or as an assistant surgeon	207 (40.3%)
Average of patients examined per week	
<30 patients	88 (17.1%)
30 - 50 patients	158 (30.7%)
51 - 100 patients	168 (32.7%)
101 - 150 patients	71 (13.8%)
151 – 200 patients	29 (05.6%)
Average of surgeries performed per week	
None	247 (48.1%)
<5	125 (24.3%)
5 - 10	95 (18.5%)
11 - 15	30 (05.8%)
16 - 20	10 (01.9%)
>20	07 (01.4%)
Average of laser treatment sessions conducted per week	
None	305 (59.3%)
<5	147 (28.6%)
5 - 10	39 (07.6%)
11 - 20	15 (02.9%)
>20	08 (01.6%)
Work-related pain	
Yes	258 (50.2%)
No	201 (39.1%)
I don’t know	55 (10.7%)
Method of treatment	
Oral medicine	131 (32.1%)
Injectable medicine	13 (03.2%)
Physiotherapy	89 (21.8%)
Combination	26 (06.4%)
None	149 (36.5%)
Physical stress level (1 – 10) (mean ± SD)	5.74 ± 2.11

The prevalence of neck, lower or upper back, and wrist and hand pain among eye care professionals is shown in Table 3. Based on the results, it was shown that the prevalence of professionals who were suffering from neck, lower or upper back, and wrist and hand pain was 66.7%. Among them, 47.9%, 40.3%, 35.6%, and 24.1% were experiencing neck pain, lower back pain, upper back pain, and wrist and hand pain, respectively. When compared between ophthalmologists versus non-ophthalmologists, it was found that the prevalence of lower back pain (X2=9.874; p=0.002) and wrist and hand pain (X2=4.752; p=0.029) were statistically significantly higher among non-ophthalmologists.

**Table 3:** Prevalence of neck, lower or upper back, and wrist and hand pain among eye care professionals

Variables	Overall N (%) ^(n=514)^	Ophtha N (%) ^(n=284)^	Non-Ophtha ^‡^ N (%) ^(n=230)^	X2	P-value ^§^
Suffering from neck, lower or upper back, wrist and hand pain	343 (66.7%)	190 (66.9%)	153 (66.5%)	0.008	0.928
Experience neck pain	246 (47.9%)	145 (51.1%)	101 (43.9%)	2.599	0.107
Experience lower back pain	207 (40.3%)	97 (34.2%)	110 (47.8%)	9.874	0.002 **
Experience upper back pain	183 (35.6%)	97 (34.2%)	86 (37.4%)	0.581	0.446
Experience wrist and hand pain	124 (24.1%)	58 (20.4%)	66 (28.7%)	4.752	0.029 **

Table 4 lists the professionals' complaints of pain mostly after work, with 84.6%, 84.1%, 83.6%, and 74.5% for wrist or hand pain, upper back pain, neck pain, and lower back pain, respectively. For frequency of pain, most professionals had a frequency of one to two times per week in the neck (35%), lower back (36.7%), upper back (27.9%), and wrist and hand pain (35.5%). In addition, the proportion of respondents who experienced moderate pain was 56.9%, 57.1%, 51.4%, and 39.5%, respectively, for neck, lower back, upper back, and wrist and hand pain.

**Table 4:** Pain characteristics of the neck, lower or upper back, and wrist and hand pain among eye care professionals

Variables	Pain			
	Neck N (%)	Lower back N (%)	Upper back N (%)	Wrist/hand N (%)
When do you experience pain in relation to working?				
Before working	02 (0.80%)	02 (01.0%)	02 (01.1%)	02 (01.7%)
After working	199 (83.6%)	149 (74.5%)	153 (84.1%)	99 (84.6%)
All time	37 (15.5%)	49 (24.5%)	27 (14.8%)	16 (13.7%)
Frequency of pain				
Daily	32 (13.0%)	42 (20.3%)	29 (15.8%)	21 (16.9%)
1 – 2 times per week	86 (35.0%)	76 (36.7%)	51 (27.9%)	44 (35.5%)
3 – 5 times per week	65 (26.4%)	57 (27.5%)	61 (33.3%)	21 (16.9%)
1 – 2 times per month	41 (16.7%)	24 (11.6%)	26 (14.2%)	22 (17.7%)
Less than once a month	22 (08.9%)	08 (03.9%)	16 (08.7%)	16 (12.9%)
Pain Severity				
Mild	95 (38.6%)	72 (35.1%)	73 (39.9%)	69 (55.6%)
Moderate	140 (56.9%)	117 (57.1%)	94 (51.4%)	49 (39.5%)
Severe	10 (04.1%)	15 (07.3%)	14 (07.7%)	04 (03.2%)
Incapacitating	01 (0.40%)	01 (0.50%)	02 (01.1%)	02 (01.6%)

When measuring the relationship between neck, lower or upper back, and wrist and hand pain among the socio-demographic characteristics of eye care professionals, it was observed that the prevalence of pain was significantly higher among females and older age groups (>50 years) (Table 5). People who are running have no difference in the prevalence of pain 48.8 and 51.2, however, 68.3% of participants who don’t do running exercises (p-value 0.011) and 92.2% of those with comorbidities (p-value 0.001) suffer from pain.

**Table 5:** Relationship between the neck, lower or upper back, and wrist and hand pain in regard to the socio-demographic characteristics of eye care professionals (n=514)

Factor	Neck, lower or upper back, wrist and hand pain		X2	P-value ^§^
	Yes ^(n=343)^ N (%)	No ^(n=171)^ N (%)		
Age group in years				
<30 years	137 (60.9%)	88 (39.1%)	6.154	0.046 **
30 – 50 years	156 (71.2%)	63 (28.8%)		
>50 years	50 (71.4%)	20 (28.6%)		
Gender				
Male	154 (57.9%)	112 (42.1%)	19.392	<0.001 **
Female	189 (76.2%)	59 (23.8%)		
Years in practice				
<5 years	143 (61.4%)	90 (38.6%)	5.622	0.060
5 – 10 years	81 (72.3%)	31 (27.7%)		
>10 years	119 (70.4%)	50 (29.6%)		
Residence region				
Central region	196 (66.0%)	101 (34.0%)	0.173	0.678
Non-Central region	147 (67.7%)	70 (32.3%)		
Occupation				
Optometrist	148 (66.1%)	76 (33.9%)	3.289	0.349
Main surgeon	146 (69.5%)	64 (30.5%)		
Assistant surgeon	44 (59.5%)	30 (40.5%)		
Orthoptist	05 (83.3%)	01 (16.7%)		
Regular exercise				
Yes	131 (66.5%)	66 (33.5%)	0.008	0.929
No	212 (66.9%)	105 (33.1%)		
Type of exercises				
Walking				
Yes	83 (61.5%)	52 (38.5%)	2.273	0.132
No	260 (68.6%)	119 (31.4%)		
Cycling				
Yes	24 (70.6%)	10 (29.4%)	0.244	0.621
No	319 (66.5%)	161 (33.5%)		
Cardio				
Yes	52 (67.5%)	25 (32.5%)	0.026	0.871
No	291 (66.6%)	146 (33.4%)		
Weightlifting				
Yes	46 (74.2%)	16 (25.8%)	1.768	0.184
No	297 (65.7%)	155 (34.3%)		
Cross fit				
Yes	17 (77.3%)	05 (22.7%)	1.150	0.283
No	326 (66.3%)	166 (33.7%)		
Boxing				
Yes	06 (66.7%)	03 (33.3%)	0.000	0.997
No	337 (66.7%)	168 (33.3%)		
Running				
Yes	20 (48.8%)	21 (51.2%)	6.467	0.011 **
No	323 (68.3%)	150 (31.7%)		
Other				
Yes	05 (62.5%)	03 (37.5%)	0.066	0.798
No	338 (66.8%)	168 (33.2%)		
BMI level				
Normal/Underweight	202 (64.1%)	113 (35.9%)	2.486	0.115
Overweight/Obese	141 (70.9%)	58 (29.1%)		
History of trauma				
Yes	72 (78.3%)	20 (21.7%)	6.709	0.010 **
No	271 (64.2%)	151 (35.8%)		
Associated comorbidities				
Yes	95 (92.2%)	08 (07.8%)	37.733	<0.001 **
No	248 (60.3%)	163 (39.7%)		
	Mean ± SD	Mean ± SD	t-test	P-value ^‡^
Physical stress level (1 – 10)	6.26 ± 1.83	4.69 ± 2.23	8.498	<0.001 **

Previous history of surgeries, type of surgeries, the average of surgeries performed per week, and the average of laser treatment sessions conducted per week were significant risk factors for pain (Table 6). Cornea surgeons have more pain (70.8%) compared to other categories of surgeons, followed by glaucoma surgeons (67.1%). Also, surgeons who perform more than 20 surgeries per week have the highest prevalence of pain. Among surgeons, 81% of them reported pain when working as main surgeons and 67.7% reported pain when working as assistant surgeons.

**Table 6:** Relationship between the neck, lower or upper back, and wrist among the previous history of surgeries and its relation to practice (n=514)

Factor	Neck, lower or upper back, and wrist and hand pain		X2	P-value ^§^
	Yes ^(n=343)^ N (%)	No ^(n=171)^ N (%)		
Previous history of surgeries				
Yes	14 (82.4%)	03 (17.6%)	1.933	0.164
No	329 (66.2%)	168 (33.8%)		
Type of surgeries				
Vitreoretinal	27 (60.0%)	18 (40.0%)	1.007	0.316
Cataract	123 (65.8%)	64 (34.2%)	0.121	0.728
Glaucoma	47 (67.1%)	23 (32.9%)	0.006	0.937
Cornea	51 (70.8%)	21 (29.2%)	0.635	0.426
Pediatric	54 (64.3%)	30 (35.7%)	0.271	0.603
Oculoplasty	36 (65.5%)	19 (34.5%)	0.045	0.832
Average of patients examined per week				
<30 patients	51 (58.0%)	37 (42.0%)	16.097	0.003 **
30 - 50 patients	92 (58.2%)	66 (41.8%)		
51 - 100 patients	124 (73.8%)	44 (26.2%)		
101 - 150 patients	55 (77.5%)	16 (22.5%)		
151 – 200 patients	21 (72.4%)	08 (27.6%)		
Average of surgeries performed/week				
<5	76 (60.8%)	49 (39.2%)	4.384	0.356
5 - 10	64 (67.4%)	31 (32.6%)		
11 - 15	23 (76.7%)	07 (23.3%)		
16 - 20	07 (70.0%)	03 (30.0%)		
>20	06 (85.7%)	01 (14.3%)		
Average of laser treatment sessions conducted per week				
<5	91 (61.9%)	56 (38.1%)	7.142	0.068
5 - 10	28 (71.8%)	11 (28.2%)		
11 - 20	12 (80.0%)	03 (20.0%)		
>20	08 (100%)	0		
Feel more pain when working as a				
Main surgeon	60 (81.1%)	14 (18.9%)	4.414	0.110
Assistant surgeon	63 (67.7%)	30 (32.3%)		
Equal severity of pain	47 (78.3%)	13 (21.7%)		

## Discussion

Musculoskeletal pain is a common issue among ophthalmologists. It is reported that more than half of ophthalmologists experience at least one type of pain, either in the lower back (39%) or neck (32.6%) [4]. Several factors have been reported that may have contributed to causing this issue among ophthalmologists. For example, working in the same posture for a long period of time, awkward posture, and bending the back [6]. Al-Ruwaili’ and Khalil’ have noted that the prevalence of lower back pain was high among ophthalmologists and healthcare professionals, which is due to sitting for a long time that ultimately increases the chance of lower back pain by 1.5 times [7-9].

Lower back pain is associated with low productivity and absenteeism [10]. Also, there is an association between work disability and musculoskeletal disorders [11]. Back pain and stress at work are related to each other [12]. Vinstrup et al. have noted that there is a positive association between stress, musculoskeletal pain, and poor quality of sleep [13], which makes us believe that the cause of pain is a cumulative effect of several known and unknown risk factors. The increased musculoskeletal pain experienced by the participants in our study may be associated with stress, anxiety, and fewer break times in the work period [14,15].

Al Shammari et al. reported that the prevalence of musculoskeletal pain increases with older age [16]. In our study, the prevalence of musculoskeletal pain was 66.7%. Among them, 47.9%, 40.3%, 35.6%, and 24.1% were experiencing neck pain, lower back pain, upper back pain, and wrist and hand pain, respectively. When looking at the demographic data of our sample, we also found that pain is more prevalent in older age (>50 years). It is also interesting that the pain was more prevalent in females. Some justify the difference by low muscle tone and strength, hormonal changes, and a higher incidence of osteoporosis among females [17]. Female dentists and nurses have shown more prevalence of neck, shoulder, and upper and lower back pain as compared to males [17,18]. Both working environments (Dentistry and Eyecare) have similarities in working conditions in the clinic where you must lean down and forward to examine or treat a patient. It was shown that there is a gender difference in the perception of pain, which may be attributed to differences in the level of psychological stress and somatic and visceral perception [19]. We think females are more likely to be sensitive to pain than males, which makes the rate of musculoskeletal pain in females higher than in males. Prudhvi and Murthy noted that obesity is related to lower back pain among dentists [20]. In our study, we also found an association between obesity and lower back pain.

We found that the prevalence of pain was higher in cornea and glaucoma surgeons. Venkatesh et al. also found that general ophthalmologists, cataract, cornea, refractive, and glaucoma surgeons, and medical retina specialists are more at risk to have back pain than pediatric ophthalmologists, neuro-ophthalmologists, oculoplastic surgeons, and retina surgeons [21]. We believe that this is also true due to the fact that pediatric ophthalmologists, neuro-ophthalmologists, oculoplastic surgeons, and retina surgeons are more dynamic in the clinic and the operating theater than other ophthalmologists. They use various types of examination equipment and methods interchangeably and are less dependent on slit lamps and surgical microscopes, which may demand a more rigid posture. Assistant surgeons are assumed to have more pain because of their awkward sitting position; however, we didn't find a significant difference in pain between main surgeons and assistant surgeons. We found that when more patients are seen each week, there is an association with a higher prevalence of pain. However, Schechet et al. noted that there was no relationship between reported pain and how many patients are seen per week [22].

To minimize the prevalence of musculoskeletal pain, several studies have recommended: moving and stretching every 10 to 15 minutes during surgery, maintaining a relaxed neutral posture, distributing the pressure equally on both foot pedals, placing the patient in a comfortable position, being closer to the bed during surgery, taking a break every 10 to 15 minutes after using the microscope, using a backrest, and having an ergonomic workplace [22,23].

Oral medicine and physiotherapy were the most common treatment options that our participants used to relieve pain. Also, they are commonly used by other healthcare providers such as otolaryngologists [24]. It was shown that using manual therapy, a form of physical therapy, along with exercising is better for neck pain than exercising or manual therapy alone [25]. A systematic review study of European back and neck pain clinical guidelines has recommended oral treatment for neck pain, however, they didn’t recommend it for lower back pain [26]. Also, correction of false posture can be important to prevent neck and arm pain [27]. Pain in our participants could be partly due to the wrong posture. There are several studies that confirm that exercise has a huge effect on lower back pain. Al Gadeeb et al. found that the prevalence of musculoskeletal pain decreases with physical activity and increases with physical inactivity [28]. Alnaami et al. noted that exercising regularly can be helpful for lower back pain, and exercising can result in a significant long-term improvement in shoulder, neck, and lower back discomfort [29,30]. In our study, participants who were not running have shown a higher prevalence of pain.

There are a few limitations in our study. We have not asked if the pain is better or relieved during vacations, which may signify more that the pain is due to work. We also should have asked surgeons if they experienced more pain after surgery as compared to the clinic. We have not included shoulder, thigh, and knee pain in the questionnaire. Other risk factors that may have contributed to pain like psychosocial factors and smoking were not asked in the questionnaire. We suggest further studies be done to investigate if there is a relationship between high working hours a week, emotional stress, and pain. The cross-sectional design of the study limits the finding of other potential risk factors.

## Conclusions

Neck, lower or upper back, and wrist and hand pain are highly prevalent among eye care professionals, as more than two-thirds of current practitioners suffer from them. This is especially true among females and older adults (>50 years). Among different exercises, running is most protective against having such pains. Comorbidities contribute significantly to developing pain. There is a higher likelihood of developing pain as the higher the average of patients examined, the more surgeries and laser sessions done per week.
